# Reproductive Status of *Onchocerca volvulus* after Ivermectin Treatment in an Ivermectin-Naïve and a Frequently Treated Population from Cameroon

**DOI:** 10.1371/journal.pntd.0002824

**Published:** 2014-04-24

**Authors:** Hugues C. Nana-Djeunga, Catherine Bourguinat, Sébastien D. Pion, Jean Bopda, Jonas A. Kengne-Ouafo, Flobert Njiokou, Roger K. Prichard, Samuel Wanji, Joseph Kamgno, Michel Boussinesq

**Affiliations:** 1 Parasitology and Ecology Laboratory, Department of Animal Biology and Physiology, Faculty of Science, University of Yaounde 1, Yaounde, Cameroon; 2 Centre for Research on Filariasis and other Tropical Diseases (CRFilMT), Yaounde, Cameroon; 3 Institute of Parasitology, McGill University, Sainte Anne-de-Bellevue, Québec, Canada; 4 UMI 233, Institut de Recherche pour le Développement (IRD) and University of Montpellier 1, Montpellier, France; 5 Research Foundation in Tropical Diseases and the Environment, Buea, Cameroon; 6 Department of Microbiology and Parasitology, University of Buea, Buea, Cameroon; 7 Faculty of Medicine and Biomedical Sciences, University of Yaounde 1, Yaounde, Cameroon; Institute of Medical Microbiology, Immunology and Parasitology, Germany

## Abstract

**Background:**

For two decades, onchocerciasis control has been based on mass treatment with ivermectin (IVM), repeated annually or six-monthly. This drug kills *Onchocerca volvulus* microfilariae (mf) present in the skin and the eyes (microfilaricidal effect) and prevents for 3–4 months the release of new mf by adult female worms (embryostatic effect). In some Ghanaian communities, the long-term use of IVM was associated with a more rapid than expected skin repopulation by mf after treatment. Here, we assessed whether the embryostatic effect of IVM on *O. volvulus* has been altered following frequent treatment in Cameroonian patients.

**Methodology:**

Onchocercal nodules were surgically removed just before (D0) and 80 days (D80) after a standard dose of IVM in two cohorts with different treatment histories: a group who had received repeated doses of IVM over 13 years, and a control group with no history of large-scale treatments. Excised nodules were digested with collagenase to isolate adult worms. Embryograms were prepared with females for the evaluation of their reproductive capacities.

**Principal Findings:**

Oocyte production was not affected by IVM. The mean number of intermediate embryos (morulae and coiled mf) decreased similarly in the two groups between D0 and D80. In contrast, an accumulation of stretched mf, either viable or degenerating, was observed at D80. However, it was observed that the increase in number of degenerating mf between D0 and D80 was much lower in the frequently treated group than in the control one (Incidence Rate Ratio: 0.25; 95% CI: 0.10–0.63; p = 0.003), which may indicate a reduced sequestration of mf in the worms from the frequently treated group.

**Conclusion/Significance:**

IVM still had an embryostatic effect on *O. volvulus*, but the effect was reduced in the frequently treated cohort compared with the control population.

## Author Summary

Onchocerciasis, also known as river blindness, is a parasitic disease due to the filarial nematode *Onchocerca volvulus*. It affects more than 37 million people worldwide, most of them (99%) living in Africa. The control of river blindness is, up to now, based on annual or six-monthly mass treatment with ivermectin. This drug kills *O. volvulus* microfilariae (mf) present in the skin and the eyes and prevents for 3–4 months the release of new mf by female worms (embryostatic effect). In Ghana, after 10–19 years of repeated treatments, the emergence of adult parasite populations not responding as expected to ivermectin was postulated. In this study, the reproductive status of female worms was compared, just before and 80 days after ivermectin treatment, between frequently treated and ivermectin-naïve cohorts from Cameroon. In both groups, embryogenesis of *O. volvulus* was not affected by ivermectin. However, the accumulation of microfilariae (mf) in the females uteri expected after ivermectin was less marked in the frequently treated population, suggesting that the temporary sequestration of mf following treatment may have been weakened in this group. After 13 years of repeated annual treatments, the embryostatic effect of ivermectin on *O. volvulus* still occurs but the present findings, associated with observations of higher rates of skin repopulation by mf in the same individuals, suggest that this effect has been decreased.

## Introduction

The macrocyclic lactone drug ivermectin (IVM) has a broad spectrum of applications against arthropods and nematodes. In human medicine, one of the major indications for IVM is the treatment of onchocerciasis or river blindness [1]. IVM targets both the microfilariae (mf) and adult stages of *Onchocerca volvulus*, the filarial nematode causing river blindness. By binding to glutamate-gated chloride (GluCl) channels, IVM may provoke pharyngeal and/or somatic paralysis of nematode parasites [2]–[5]. In *Brugia malayi*, a filarial nematode closely related to *O. volvulus*, it has been postulated that IVM may paralyze the muscle associated with the excretory vesicle, leading to a reduction in the release of immunomodulators from the parasite that enable evasion of the host immune system [6]. In synergy with the host immune response, this paralyzing effect possibly leads to the elimination of *O. volvulus* skin mf. Following a standard therapeutic dose (150 µg/kg of bodyweight), this so-called microfilaricidal effect of IVM leads to a 98% clearance of the skin mf within 2–3 weeks [7]. However, a standard dose of IVM is not adulticidal for *O. volvulus* even though repeated treatments at short intervals (≤3 months) have a significant effect on the viability of a proportion of adult worms [8]. The effect of IVM on adult male worms is not very well known but multiple doses may reduce their ability to re-inseminate the females [9]. In female worms, the drug prevents, temporarily, the release of mf from the uteri. Apparently, IVM has no effect on the embryogenesis *per se*, but the newly produced mf accumulate in the uteri and degenerate *in situ*. This is the so-called embryostatic effect of IVM. This inhibition of release of viable mf for some months is important, together with the initial microfilaricidal effect, for reducing the transmission of the parasite.

Because of the longevity of adult worms, IVM distribution programs need to be sustained for 15–20 years, with a high level of suppression of parasite transmission, if one wants to reach elimination of the parasite in the population [10]–[13]. Unfortunately, suboptimal responses to IVM have been reported from some Ghanaian communities that had been subjected to 10–19 rounds of annual community treatments [14], [15]. In those poorly responding communities, repopulation of the skin by mf after IVM was unexpectedly rapid in a fraction of the population, and the response to IVM by *O. volvulus* was considered atypical [16]. Indeed, the joint analysis of skin microfilarial dynamics after treatment and of female adult worms' reproductive capacity suggests that in these Ghanaian communities, the strength of the embryostatic effect of IVM has been reduced in some parasites that had been previously exposed repeatedly to this drug.

In a previous paper, we compared the dynamics of *O. volvulus* skin microfilarial densities after IVM treatment in two cohorts with contrasting exposure to this drug: one which had received repeated treatment for 13 years and one which had no history of large-scale treatments. We observed that the repopulation rate was significantly higher in the frequently treated group than in the controls between 15 and 80 days post-IVM, which suggests that the worms from the frequently treated area had resumed their capacity to release mf earlier [17]. In the present paper, we analyzed the reproductive status of *O. volvulus* female worms collected and the composition of the different embryonic stages found in utero, in the same cohorts before (D0) and 80 days (D80) after IVM, to assess whether embryo production, development and viability in the females are consistent with our previous findings.

## Methods

### Study design and selection of patients

The objective of this study was to assess whether the embryo production and development, and/or the embryostatic effect of IVM on *O. volvulus* have been altered after several years of drug pressure. To do this, we composed two cohorts of patients and defined the exposure factor as the area of residence (frequently treated area or area naïve to mass IVM administration).

The group from the IVM-naïve area was recruited in 10 neighboring communities of the Nkam valley (Bayon, Ekom-Nkam, Mboue, Mpaka, Mbarembeng, Bakem 1, Bakem 2, Lonze, Manjibo and Mounko), a forested area located in the Littoral Region of Cameroon. These villages were known to be endemic for onchocerciasis but had not benefitted from any mass IVM treatment at the outset of the study. Since the IVM-naïve region was also known to be endemic for loiasis, *Loa loa* microfilaremia was assessed and those few subjects presenting with more than 30,000 *L. loa* mf per milliliter of blood were excluded from the study to prevent the occurrence of a *Loa*-related post-IVM encephalopathy. This IVM-naïve area will be referred, throughout the text, as the control area.

The group of patients subjected to multiple IVM treatments was recruited in 22 communities of the Mbam valley (Babetta, Balamba 1, Balamba 2, Bayomen, Bialanguena, Biamo, Biatsotta, Boalondo, Bombatto, Botatango, Boura 1, Diodaré, Gah-Bapé, Kalong, Kon, Lablé, Lakpang, Ngomo, Ngongol, Nyamanga, Nyamsong and Yébékolo). In these communities, annual large-scale treatments with IVM have been conducted since 1994. In addition, these patients had taken part in a clinical trial conducted between 1994 and 1997 aimed at evaluating the macrofilaricidal potential of IVM [8]. During this clinical trial, eligible patients were randomly allocated to one of the four following IVM treatment groups: 150 µg/kg body weight annually (standard group or group 1); 150 µg/kg three-monthly (group 2); high doses (one dose of 400 µg/kg and then two doses of 800 µg/kg) annually (group 3); and high doses (two doses of 400 µg/kg and then 10 doses of 800 µg/kg) three-monthly (group 4). A “clearing dose” of IVM (150 µg/kg) was given to all volunteers in May 1994 to avoid the possibility of severe reactions developing in any patients subsequently taking their first dose on the high-dose regimen and the first “trial” treatment was given three months later. Thus, over the four-year study period (1994–1997) and depending on their treatment group during the trial, they received 4 to 13 doses of IVM under the direct observation of the investigators.

To date, no vector control has ever been implemented in either study area.

Patients eligible for the present study, either from the control or the frequently treated area, were males aged 25 years and over carrying at least two palpable onchocercal nodules, but otherwise in a good state of health. All eligible subjects, including those from the IVM-naïve area, were questioned about their history of IVM treatment. A small number of patients from the IVM-naïve area declared they had occasionally received the drug during distribution campaigns organized in communities located 10–20 km away, in the West Region where large scale treatments with IVM had been ongoing for more than 10 years. Assuming that the effect of IVM on adult worm reproduction gradually disappears after 9 months [18], all individuals who had taken IVM during the previous 9 months were discarded from the analysis. Consequently, the effect of a single dose of IVM, and not the potential cumulative effect of two doses of IVM given within a short time frame was assessed. A total of 15 individuals from the frequently treated population declared having taken IVM more recently than the previous Community-Directed Treatment with IVM which had taken place about 9 months before the first nodulectomy planned for the present study, and were thus excluded from the analyses.

To assess whether the embryostatic effect of IVM had been reduced in the frequently treated population, the reproductive activity of *O. volvulus* adult female worms was evaluated in both populations before and 80 days after the administration of IVM.

### Ethical clearance and agreement

The study received ethical clearance from the National Ethics Committee of Cameroon and was approved by the Cameroonian Ministry of Public Health. The objectives and schedule of the study were explained to all eligible individuals, and those who agreed to participate signed a consent form and kept a copy of the latter.

### Processing of nodules, isolation and examination of adult worms

The diagnosis and extirpation of onchocercal nodules were performed as previously described [19], with only slight modifications. Briefly, subcutaneous nodules were sought, at the outset of the study, by visual inspection of subjects, then by careful palpation in a closed but well illuminated room. The locations of all palpated nodules were recorded on a body chart. Two of these locations were randomly selected for subsequent surgical removal, the first one just before the administration of IVM, and the second one 80 days after treatment. Nodulectomies were performed under optimal aseptic conditions. All nodules present in the randomly chosen anatomical sites were collected and each was placed individually in a Petri dish containing RPMI-1640 medium (GIBCO, Life Technologies Inc., Burlington, ON, Canada) in which they were cleaned of remaining human tissue. The nodules were then stored in liquid nitrogen until use.

In order to isolate the adult worms contained in the nodules, the latter were digested using the collagenase technique [20]–[22], blinded as to their origin (frequently treated or IVM-naïve area), or date of nodulectomy (pre- or post-treatment). After thawing, each nodule was incubated for 12–19 hours at 35°C or 37°C (time and temperature of digestion depending on the nodule's weight) in five milliliters of the culture medium 199 (GIBCO, Life Technologies Inc., Burlington, ON, Canada) containing type I collagenase (SIGMA, Aldrich Co., Oakville, ON, Canada) at a final concentration of 2.25 mg/ml. Details on the process of the nodule digestion are given as supplementary information (Text S1). The product of digestion (the worm mass and digested human tissues constituting the nodule) was placed in a Petri dish containing 15 ml of medium 199 enriched with Earle's salts (E199), L-glutamine, sodium bicarbonate (GIBCO, Life Technologies Inc., Burlington, ON, Canada), and supplemented with gentamicin sulfate (SIGMA, Aldrich Co., Oakville, ON, Canada) at a final concentration of 2 mg/ml. Individual worms were isolated under a dissecting microscope using entomological and Dumont #5 forceps (Fine Science Tools GmbH, Heidelberg, Germany). Each entire and live worm (dead or calcified and incomplete or broken worms were counted but discarded from the further process) was then spread on a labeled slide and examined under a light microscope (magnification ×40) to confirm the sex of the worm. Entire male worms were individually frozen for subsequent genotyping. In the case of female worms, the head and the tail were localized, and the whole worm examined to determine whether it had been broken during the isolation process. A 15 mm-long section was then removed with a scalpel from the tail end, of each complete and unbroken female, for subsequent genotyping. The rest of the body of the female worm was used to prepare embryograms: it was cut in 1 mm thin slices and crushed in a porcelain mortar containing 1 ml of medium 199. To avoid the shells of embryos breaking during the crushing process, the mortar was placed on a 3 cm thick wet sponge to absorb shocks between the pestle (also in porcelain) and the mortar. Fifteen microliters of the homogenized resulting suspension was then transferred into a 0.2 mm deep Malassez counting chamber and the embryograms were examined under a light microscope (magnification ×100 or ×400).

All embryonic stages were identified and counted according to the following classification: viable stretched mf, degenerating stretched mf, viable coiled mf, degenerating coiled mf, viable morulae and degenerating morulae [21]. The density of oocytes was assessed in a semi-quantitative manner using four categories: absence, rare (less than one oocyte per square of the counting chamber or PSC), few (1–10 oocytes PSC) and numerous (more than 10 oocytes PSC). The suspensions with embryos were examined by two experienced and independent investigators and when any discrepancy was found, the preparation was re-examined by both investigators.

The evaluation of the uterine content was made from 15 µl of the homogenized suspension resulting from the crushing of each female worm; for a matter of simplicity, we shall express the numbers of embryos using this volume (15 µl) as arbitrary unit.

### Statistical analysis

The reproductive status of the female worms was analyzed using one qualitative and three quantitative criteria.

#### Qualitative aspects

Following the definition proposed by Kläger and colleagues [23], we considered as “productive” any live female (neither dead, nor calcified) presenting with an active embryogram, *i.e.* all worms whose embryogram contained any viable (not degenerating) embryonic stages (morulae and/or coiled mf and/or stretched mf). Thus, worms containing only degenerating embryos were not taken into account in the analyses of productive worms. The proportions of “productive” worms were first compared between the sites using simple chi-squared tests. Then, to account for the hierarchical structure of the data (several worms from a same nodule, several nodules from a same individual) the comparisons were performed using a multilevel (random-effects) logistic regression (see Text S2 in supporting information for explicit specification). Significance of the random effects was tested using log-likelihood tests.

#### Quantitative aspects

The reproductive status was also analyzed according to three quantitative indicators: the mean number of all embryos (morulae, coiled and stretched mf, either viable or degenerating) per worm, the mean number of viable mf per worm and the mean number of degenerating mf per worm.

The distributions of these quantitative indicators among female worms were compared between the sites using the Kolmogorov-Smirnov test. Random-effect Poisson regression models were used to compare the quantitative criteria between the two groups while accounting for the data structure.

#### Adjustment factors

It has been shown that *O. volvulus* skin microfilarial density, assessed after IVM treatment, is associated with host factors, such as age and the number of palpable nodules [24]. Assuming that such host-factor influences could also be expected at the worm level, the following cofactors were included in the regression models as adjustment covariates: host age (continuous variable), total number of nodules found at palpation (continuous variable), anatomic site at which the nodule was collected (categorical: (1) head and upper limbs; (2) thorax and trunk; (3) iliac crests; (4) greater trochanters; and (5) knees and legs), total number of nodules present in (and excised from) the nodulectomy site, total number of female worms in the nodule, and total number of male worms in the nodule. In addition, we included an interaction term between the study group and the number of days after IVM treatment (0 or 80) to compare, between the two groups, the effect of the drug on the reproductive status of the worms.

Logistic (on qualitative indicators) or Poisson (on quantitative indicators) regression likelihood methods were applied to control possible confounders and to identify cofactors associated with the dependent variable. Significance of the random-effect parameters was tested using likelihood ratio tests.

## Results

### Description of biological samples collected and processed

In the control group, 190 individuals underwent nodulectomies before receiving IVM, and 171 (90.0%) of them were present for the second round of nodulectomy, 80 days later. One hundred and eighty eight frequently treated individuals underwent nodulectomies before receiving IVM, and 159 (84.6%) of them took part in the second round of nodulectomy. Overall, the 708 surgical interventions led to the collection of 1110 nodules, of which 1069 were examined and contained 1230 male and 2036 female worms (Table 1). Details on the composition of the nodules for each study site and time of examination are given in Table 1.

**Table 1:** Composition of the nodules and distribution of female worms according to their uterine contents.

Study area/period	Control D0	Frequently treated D0	Control D80	Frequently treated D80
Number of individuals who provided ≥1 nodule	190	188	171	159
Total number of nodules excised	296	291	269	254
Total number of nodules examined	279	285	262	243
Mean number of nodules examined per individual (sd)	1.56 (1.37)	1.55 (1.04)	1.57 (0.86)	1.60 (0.97)
Total number of female worms in the nodules	568	580	487	401
Mean number of female worms per nodule (sd)	2.04 (1.77)	2.04 (1.79)	1.86 (1.41)	1.65 (1.43)
Mean number of female worms per patient (sd)	2.29 (1.94)	2.28 (1.94)	2.09 (1.55)	1.87 (1.64)
Total number of dead or calcified female worms in the nodules	32	38	42	40
Total number of incomplete or broken female worms in the nodules	67	71	49	41
Total number of complete and live female worms in the nodules	469	471	396	320
Total number of male worms in the nodules	384	346	271	229
Mean number of male worms per nodule (sd)	1.38 (1.75)	1.21 (1.69)	1.03 (1.07)	0.94 (1.31)
Mean number of male worms per patient (sd)	1.57 (1.98)	1.34 (1.85)	1.16 (1.11)	1 (1.38)
Number of individuals for which ≥1 worm had an embryogram	165	153	147	128
Number of nodules for which ≥1 worm had an embryogram	223	210	205	178
Number of female worms for which an embryogram was prepared	469	471	396	320
% of productive female worms	49.3	45.3	43.2	41.5
Number of embryos per female worm (mean/sd/max)	54.9/97.0/645	54.3/90.3/700	72.4/185.7/1364	72.5/160.9/1750
Number of viable morulae per female worm (mean/sd/max)	17.3/38.6/310	12.5/30.8/325	5.6/19.4/230	7.7/30.5/340
Number of viable coiled mf per female worm (mean/sd/max)	15.0/36.9/336	12.2/30.0/245	4.7/17.9/205	6.3/21.2/190
Number of embryos per productive female worm (mean/sd/max)	102.2/116.8/645	103.0/106.5/700	156.5/252.5/1364	154.1/220.4/1750
Number of viable morulae per productive female worm (mean/sd/max)	35.1/49.0/310	27.8/41.1/325	13.0/28.0/230	18.8/45.4/340
Number of viable coiled mf per productive female worm (mean/sd/max)	30.4/47.9/336	27.0/40.0/245	11.0/26.1/205	15.2/31.0/190
% of female worms with viable stretched mf	45.2	38.7	37.4	34.7
Number of viable stretched mf per female worm (mean/sd/max)	12.6/24.9/160	8.5/19.7/130	17.1/80.0/1250	11.4/44.6/450
% of female worms with degenerating stretched mf	31.6	48.7	47.9	58.1
Number of degenerating stretched mf per female worm (mean/sd/max)	5.4/22.7/330	16.4/45.6/430	42.2/134.6/1200	42.2/110.7/1100
% of female worms with viable or degenerating stretched mf	58.6	62.4	57.8	63.1

The evaluation of the uterine content was done from 15 µl of the homogenized suspension resulting from the crushing of each female worm. We expressed the numbers of embryos using this volume (15 µl) as arbitrary unit; sd: standard deviation; mf: microfilariae; max: maximum.

At D0, embryograms were performed on 469 and 471 female worms from the control and the frequently treated groups, respectively, and at D80, embryograms were done on 396 and 320 worms from the control and the frequently treated groups, respectively. Thus embryograms were available for 1656 of the 2036 females isolated, the difference consisting of incomplete or broken worms and of dead or calcified worms (Table 1). The distribution of female worms according to the contents of their uteri is summarized in Table 1.

### Oocyte and intermediate stages (morulae and coiled mf) production

**Figure 1:**
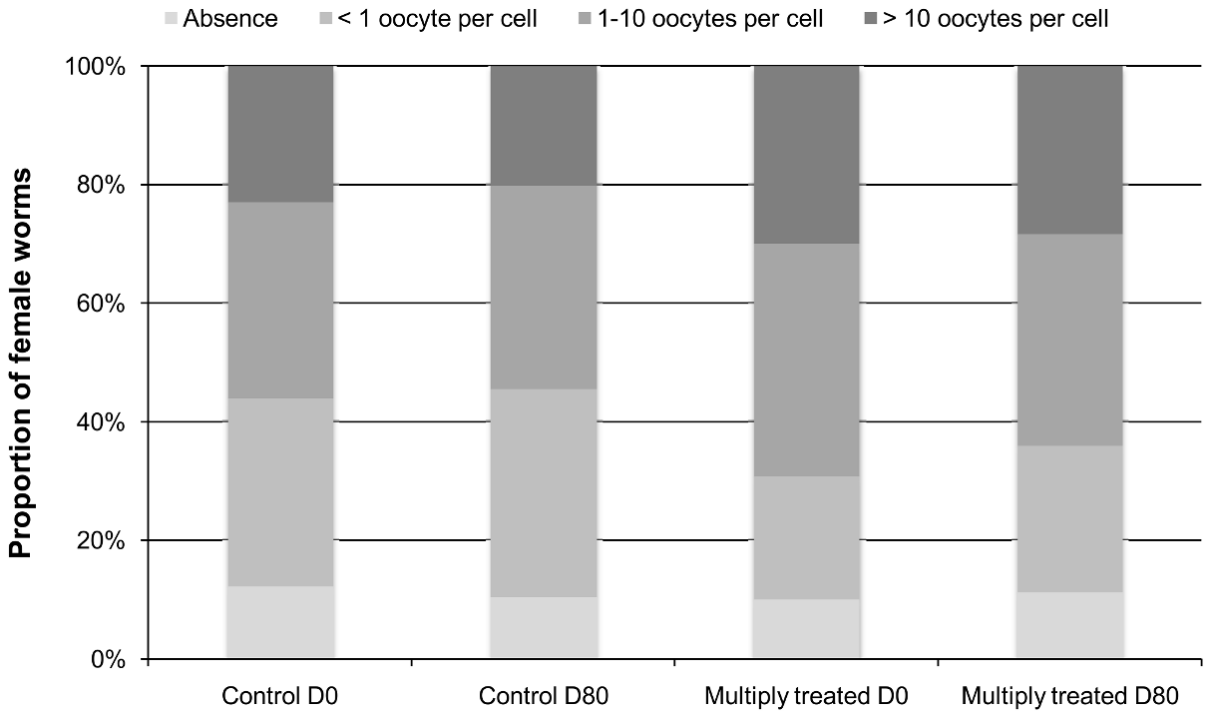
Distribution of female worms according to the density of oocytes in their uteri. A semi-quantitative approach was used to classify the density of oocytes into four categories: absence, rare (less than one PSC), few (1–10 oocytes PSC) and numerous (more than 10 oocytes PSC). The density of oocytes was assessed from 15 µl of the homogenized suspension resulting from the crushing of each female worm.

Before IVM, most worms contained oocytes (87.8% in the control group *vs* 90.0% in the frequently treated). In the frequently treated group, female worms appeared to have a slightly higher oocyte production than in the control group, with higher proportions of females containing oocytes density of 1–10 oocytes PSC and >10 oocytes PSC (Chi-squared: 18.509; 3 degrees of freedom (df); p = 0.0003) (Figure 1). This difference remained at D80 (Chi-squared: 11.544; p = 0.0091). However, in both the control and frequently treated groups, the distribution of worms according to their oocyte production did not change between D0 and D80 (Chi-squared: 2.396; 3 df; p = 0.4943 and Chi-squared: 2.226; 3 df; p = 0.5268, respectively), meaning that oocyte production remained unchanged after the IVM dose given as part of the study.

**Figure 2:**
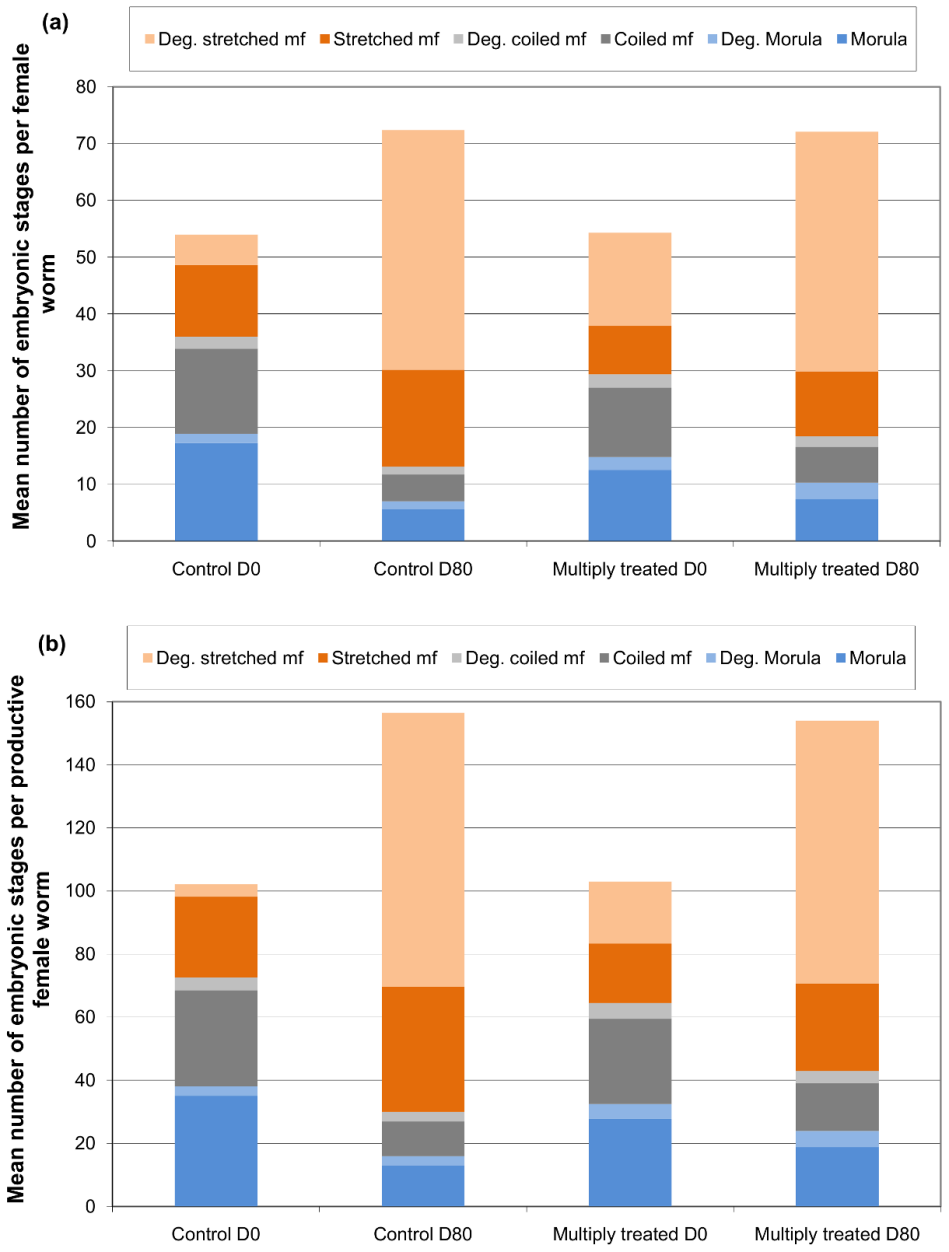
Mean number of embryos per female worm before and 80 days after ivermectin treatment. These data are presented separately for all female worms (a) and only for productive female worms (b) and are compared between the control and the frequently ( = multiply) treated groups. The mean number of embryos was assessed from 15 µl of the homogenized suspension resulting from the crushing of each female worm.

A reduction in the mean number of viable morulae per female worm was observed between D0 and D80 in the two groups (Table 1, Figure 2a). This reduction was less marked in the frequently treated group (38.4% decrease) than in the control group (67.6% decrease). A very similar pattern was observed for the mean number of viable coiled mf per female worm, with an average decrease of 32.4% in the frequently treated group and 68.7% in the control group (Table 1, Figure 2a).

### Total number of embryos, whatever the stage and the viability, in all female worms

The mean numbers of embryos per worm are summarized in Figure 2a for each group and for each time of observation. Similar numbers of embryos per worm were observed in the two groups both before IVM (mean (standard deviation, sd): 54.9 (97.0) in the control group *vs* 54.3 (90.3) in the frequently treated group) and 80 days after IVM (mean (sd): 72.4 (185.7) in the control group *vs* 72.5 (160.9) in the frequently treated group) (Table 1). Multilevel Poisson regression confirmed a similar evolution in the total number of embryos per worm in the two groups between D0 and D80 (incidence rate ratio, IRR: 0.67; 95% Confidence Interval (95% CI): 0.28–1.61; p = 0.37) (Table S1). The number of males present in the nodule was the only covariate associated with the number of embryos per worm (IRR: 2.10; 95% CI: 1.77–2.49; p = 0.001).

### Proportion of productive female worms

The mean numbers of embryos per productive worm are summarized in Figure 2b for each group and for each time of observation. At D0, 49.3% of the worms from the control group were productive with an average of 102.2 (sd: 116.8) embryos (viable or degenerating) per productive worm (Table 1). In the frequently treated group, we observed similar values, with 45.3% of productive worms and an average of 103.0 (sd: 106.5) embryos (viable or degenerating) per productive worm. At D80, the proportion of productive females decreased slightly in the two groups to reach 43.2% in the control group and to 41.5% in the frequently treated group. On average, at D80, the productive females from the control and frequently treated groups contained 156.5 (sd: 252.5) and 154.1 (sd: 220.4) viable or degenerate embryos per worm, respectively (Table 1). Multilevel logistic regression of the productive status of female worms showed an absence of significant difference between the two groups at each nodulectomy round and that changes in the proportion of productive worms between D0 and D80 were similar in the two groups (OR: 0.97; 95% CI: 0.50–1.26; p = 0.339) (Table S2). The number of male worms in the nodule was the only covariate significantly associated with the productive status of female worms.

### Number of viable stretched microfilariae in all female worms

At D0, the proportion of female worms with viable stretched mf was significantly higher in the control group than in the frequently treated group (45.2% *vs* 38.7%, respectively, p = 0.044) (Table 1). At D80, these proportions had slightly decreased and were not anymore significantly different between the two groups (37.4% *vs* 34.7% in the control and frequently treated group, respectively, p = 0.45). Similarly, before treatment, the number of viable stretched mf per worm (all worms) was slightly higher in the controls than in the frequently treated group (mean (sd): 12.6 (24.9) *vs* 8.5 (19.7), respectively, p = 0.002). The number of viable stretched mf per worm increased by about 35% in both groups at D80 (mean (sd): 17.1 (80.0) *vs* 11.4 (44.6) in the control and frequently treated group, respectively, p = 0.127) (Figure 2a). Multilevel Poisson regression did not show a significant difference between the two groups in the evolution of number of viable stretched mf per worm from D0 to D80 (IRR: 1.03; 95% CI: 0.37–2.89; p = 0.949) (Table S3). The numbers of male and of female worms in the nodule were positively and significantly associated with the number of viable stretched mf per worm (p = 0.001 and 0.015, respectively).

### Number of degenerating stretched microfilariae in all female worms

**Figure 3:**
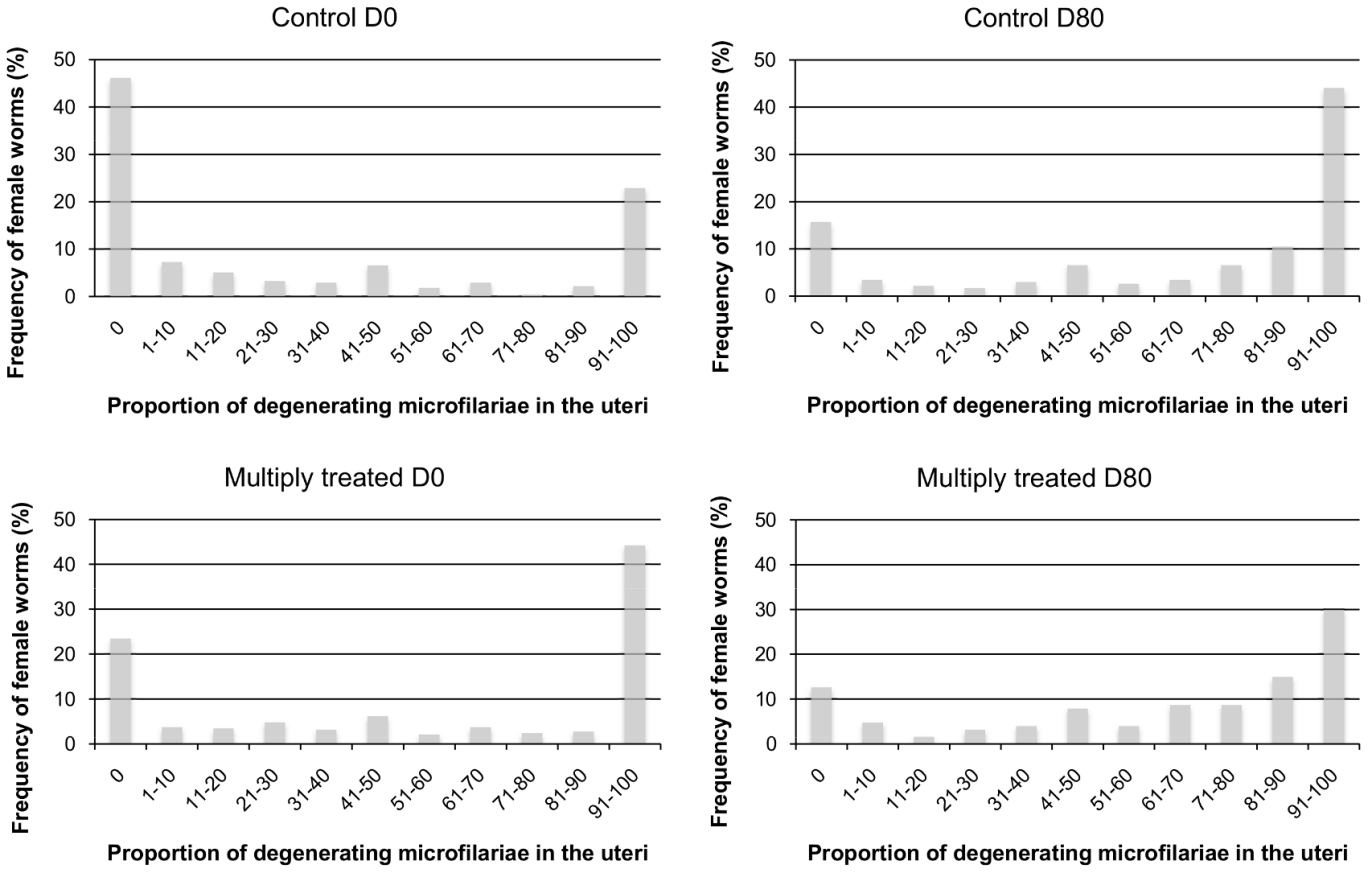
Frequency of female worms as a function of the proportion of degenerating microfilariae in their uteri. These frequencies were plotted before and 80 days after ivermectin treatment for the control and frequently ( = multiply) treated groups. The proportion of degenerating microfilariae was assessed from 15 µl of the homogenized suspension resulting from the crushing of each female worm.

At D0, the proportion of females with degenerating stretched mf was significantly higher in the frequently treated than in the control group (48.7% *vs* 31.6%, respectively, p<0.001) (Table 1). This was associated with a higher number of degenerating stretched mf per worm (all worms) in the frequently treated group (mean (sd): 16.4 (45.6)) than in the control group (mean (sd): 5.4 (22.7)) (Figures 2a and 3). At D80, the proportion of females with degenerating stretched mf was still higher in the frequently treated group (58.1% *vs* 47.9% in the control group) but the mean number of degenerating stretched mf per worm was the same in the two groups (mean (sd): 42.2 (110.7) *vs* 42.2 (134.6) in the frequently treated and the control group, respectively) (Figures 2a and 3). However, the multilevel Poisson regression indicated that the increase in number of degenerating stretched mf per worm between D0 and D80 was much lower in the frequently treated group than in controls (IRR: 0.25; 95% CI: 0.10–0.63; p = 0.003) (Table S4). Moreover, it showed that age (IRR: 1.02; 95% CI: 1.00–1.04; p = 0.038) and the number of male worms in the nodules (IRR: 2.08; 95% CI: 1.75–2.48; p = 0.001) were positively associated with the number of degenerating stretched mf.

### Summary of observations

Oocyte production was unchanged after IVM treatment in both groups. The proportion of productive females was slightly reduced after IVM in both groups but the uteri of those productive females contained about 50% more embryos, all stages considered together, than before treatment. Whereas the numbers of morulae and coiled mf both decreased after IVM, especially in the control group, the number of viable mf increased significantly (by about 35%) in both groups. The number of degenerating mf in the uteri of the worms also increased after IVM in both groups, but this accumulation was more marked in the worms from the control group.

## Discussion

The present study was carried out in a context where many controversies about possible resistance of *O. volvulus* to IVM still subsist [25]–[28]. As a chapter of a detailed study conducted in Cameroon to address this issue, this investigation aimed at assessing whether the strength of the embryostatic effect of IVM against the parasite has been modified after repeated treatments. To this end, we compared the embryonic populations, before and 80 days after a standard dose of IVM, between worms collected from naïve and frequently treated cohorts of Cameroonians.

In the design of the study, we tried to match the two groups as much as possible, except for the history of drug administration, on all other factors related to the epidemiology of onchocerciasis (age, sex, level of endemicity of river blindness, *Simulium* species, human activities, individual level of infection). Yet, to account for residual differences between the groups, these individual host factors were included as adjustment covariates in the regression models (either Poisson or logistic) while comparing the effect of IVM on embryonic populations between the two groups.

Embryograms revealed that the worms from the repeatedly treated cohort had a higher oocyte production compared to the naïve worms, suggesting that the former may have a higher capacity of reproduction than the latter. Nonetheless, at D80, the oocyte production was similar to its level at D0 in the two groups. These results confirm that oocyte production is not affected by IVM [29].

Morulae and coiled mf were also found at D80, which confirms that IVM does not interrupt the embryogenesis of *O. volvulus* [18], [30]. However, despite the unchanged production of oocytes after IVM treatment (Figure 1), we observed a reduction in the mean number of viable morulae and coiled mf per female worm between D0 and D80 (Figure 2a and 2b). Such a reduction has been previously described in *O. volvulus* [30] and *Dirofilaria immitis* (dog heartworm) [31]. Maintenance of oocyte production associated with a reduction of morulae and coiled mf suggests that the oocytes were likely not fertilized after treatment, probably due to a lack of female re-insemination [9], [32]. It has been hypothesized that IVM interferes with mate-finding by reducing the number of male worms in the nodules [33]. Migration of male worms away from the nodules might be due to the fact that IVM concentration is higher in the latter than in other human host tissues [34]–[36]. In view of the probable effect of IVM on release of substances from the excretory pore of filariae [6], one could alternatively hypothesize that IVM may block the release of sex pheromones from the female worms which normally attract male worms to the nodule and to mate with the female worms. Investigating the effects of multiple monthly doses of IVM on adult *O. volvulus*, Duke *et al.* [37] also provided histological evidences that, after IVM, sperm of male worms can be stuck in the mass of degenerating mf in the anterior parts of the uteri of re-inseminated female worms. This suggests that, despite re-insemination, the sperm would be unable to reach the seminal receptacle of a proportion of female worms.

In the present study, an accumulation of stretched mf (either viable or degenerating) in female worms uteri was observed in both groups after IVM treatment. This indicates that the embryostatic effect of IVM was still operating in the worms from the frequently treated population. However, and this is probably the most interesting finding of our study, we observed a much lower increase in the mean number of degenerating stretched mf between D0 and D80 in the frequently treated cohort compared to the control group. The physiological mechanisms associated with degenerative changes of *O. volvulus* mf in utero have not been elucidated. In the skin, degeneration of mf results from immunological process induced or facilitated by IVM [38]–[40]. However, in the uteri, mf are not in contact with the host immune cells. As suggested by recent observations on *B. malayi*, IVM might prevent the release of mature mf by interacting with glutamate-gated chloride channels localized in the uterine wall [5]. A prolonged stay in the uterus may not be suitable to mf survival, especially when they are densely packed and, as an indirect consequence of IVM, sequestrated mf may degenerate quicker than those living in their natural environment, the dermis. The lower increase in the number of degenerating mf in those worms repeatedly exposed to the drug might thus reflect an earlier than expected weakening of the embryostatic effect of IVM, allowing viable mf to move from the uteri. The genetic characterization of the worms collected as part of this study, using genes associated with the mode of action of IVM such as the avr-14 gene coding for GluCl [5], [41], are warranted to confirm possible selection towards resistance. Precisely, correlation between embryogram results and genetic profile of these worms will be particularly informative to assess whether some worms have become less sensitive to IVM, and in which proportion.

A limitation of our study may be related to the observation that, despite matching the two study groups on a number of criteria, a higher mean number of degenerating stretched mf was observed at D0, i.e. about 9 months after the last distribution of IVM in the frequently treated population, in the worms from the latter group as compared to the control group. This might be explained by a cumulative effect of repeated IVM treatments on the uteri wall. This could also be the consequence of a different age structure in the worm population between the two areas. It has been shown that, in areas of the former Onchocerciasis Control Programme in West Africa, a sustained decrease in transmission brings about an ageing of the worm population [42], associated with an increase in the proportion of old female worms harboring degenerating stretched mf [18]. The mean age of the parasites in the frequently treated population is probably higher following the decrease in transmission in this area where large-scale IVM treatments have been ongoing for more than 10 years [43]. However, since we did not score the adult worms for age, we cannot assess the respective roles of previous IVM distributions and of a possible ageing of the worm population on the excess of degenerating mf in the frequently treated group at D0. This being said, we do not think that this difference at D0 may have influenced the effect of the IVM dose given during the study.

As an ancillary result of our analyses, a positive association was observed between the number of female worms in a nodule and the number of viable stretched mf observed in their uteri. This might be explained by a stronger effect of grouped female worms to attract male worms for mating and insemination. In *Nippostrongylus brasiliensis* and *Trichinella spiralis*, a strong dosage-dependency to female pheromone was observed in male worms [44]–[47]. This means that the higher the number of female worms, the higher the number of male worms attracted and consequently the higher the chance of mating. The influence of pheromone produced by female worms in the attractiveness of male worms was considered in *O. volvulus* [29]. In the present study, the number of male worms in a nodule was also positively associated with the number of viable stretched mf observed in the female worms' uteri, indicating that the oocyte fertilization succeeded for a proportion of female worms in those nodules with higher number of female and male worms.

The present study demonstrated that the embryostatic effect of IVM on *O. volvulus* was still present even after multiple treatments. Nevertheless, this effect appears to weaken earlier after treatment in the frequently treated cohort. The higher repopulation rate of the skin by mf after IVM treatment in the individuals from the frequently treated area is consistent with an earlier recovery of mf productivity of their worms [17]. Genetic selection has been described in worm populations submitted to a high drug pressure, including worms collected from individuals of the frequently treated group of the present study [48]–[50]. The analysis of the genetic profile of the adult worms, mf and infective larvae collected as part of this study would constitute the last piece of the puzzle to complete these investigations.

## Supporting Information

Table S1 Comparison of the changes in the total number of embryos observed in the uteri of all female worms between control and frequently (multiply) treated cohorts. A Poisson regression model was used to assess the evolution between D0 and D80.
(DOC)

Table S2 Comparison of the changes in the productive status of female worms between control and frequently (multiply) treated cohorts. A logistic regression model was used to assess the evolution between D0 and D80.
(DOC)

Table S3 Comparison of the changes in the number of viable stretched mf observed in the uteri of female worms between control and frequently (multiply) treated cohorts. A Poisson regression model was used to assess the evolution between D0 and D80.
(DOC)

Table S4 Comparison of the changes in the number of degenerating stretched mf observed in the uteri of female worms between control and frequently (multiply) treated cohorts. A Poisson regression model was used to assess the evolution between D0 and D80.
(DOC)

Text S1 Procedure for the digestion of nodules to isolate *Onchocerca volvulus* worms.
(DOC)

Text S2 Formulation of the mathematical model used for data analyses.
(DOC)
