# Supplementary material for: Reproductive Status of Onchocerca volvulus after Ivermectin Treatment in an Ivermectin-Naïve and a Frequently Treated Population from Cameroon
Source: PLoS Negl Trop Dis. 2014 Apr 24;8(4):e2824. doi: 10.1371/journal.pntd.0002824 (PMC3998936; doi:10.1371/journal.pntd.0002824)
Supplement: Table S2 — Comparison of the changes in the productive status of female worms between control and frequently (multiply) treated cohorts. A logistic regression model was used to assess the evolution between D0 and D80. (DOC) [file pntd.0002824.s002.doc]

**Supporting information**

**Table S2.doc: Comparison of the changes in the productive status of female worms between control and frequently (multiply)** treated cohorts. A logistic regression model was used to assess the evolution between D0 and D80

|  | Odds-ratio | [95% Confidence Interval] | | | p-value |
| --- | --- | --- | --- | --- | --- |
| Date of follow-up |  |  |  |  |  |
| Day 0 | 1 |  |  |  |  |
| Day 80 | 0.80 | 0.51 | - | 1.26 | 0.339 |
| Study group |  |  |  |  |  |
| Control | 1 |  |  |  |  |
| Multiply treated | 0.68 | 0.41 | - | 1.12 | 0.128 |
| Date x Study group |  |  |  |  |  |
| Day 0 x Control | 1 |  |  |  |  |
| Day 80 x Multiply treated | 0.97 | 0.50 | - | 1.26 | 0.339 |
|  |  |  |  |  |  |
| Age | 0.99 | 0.97 | - | 1.00 | 0.133 |
| Number of palpable nodules | 1.05 | 0.95 | - | 1.16 | 0.354 |
|  |  |  |  |  |  |
| Anatomic site (nodulectomy) |  |  |  |  |  |
| Head and upper limbs | 1 |  |  |  |  |
| Thorax | 0.59 | 0.30 | - | 1.12 | 0.107 |
| Iliac crests | 1.00 | 0.59 | - | 1.71 | 0.993 |
| Greater trochanters | 0.85 | 0.47 | - | 1.51 | 0.568 |
| Knees and legs | 0.64 | 0.35 | - | 1.17 | 0.147 |
|  |  |  |  |  |  |
| Number of female worms | 0.98 | 0.88 | - | 1.09 | 0.671 |
| Number of male worms | 1.19 | 1.05 | - | 1.34 | 0.005 |
|  |  |  |  |  |  |
| Constant | 0.469 | 0.150 | - | 1.147 | 0.194 |
|  |  |  |  |  |  |
| Random-effect parameters |  |  |  |  |  |
| Individual level | 0.70 | 0.43 | - | 1.13 |  |
| Nodule level | 1.36 | 1.04 | - | 1.77 |  |
