# Supplementary material for: Reproductive Status of Onchocerca volvulus after Ivermectin Treatment in an Ivermectin-Naïve and a Frequently Treated Population from Cameroon
Source: PLoS Negl Trop Dis. 2014 Apr 24;8(4):e2824. doi: 10.1371/journal.pntd.0002824 (PMC3998936; doi:10.1371/journal.pntd.0002824)
Supplement: Table S3 — Comparison of the changes in the number of viable stretched mf observed in the uteri of female worms between control and frequently (multiply) treated cohorts. A Poisson regression model was used to assess the evolution between D0 and D80. (DOC) [file pntd.0002824.s003.doc]

**Supporting information**

**Table S3.doc: Comparison of the changes in the number of viable stretched mf observed in the uteri of female worms between control and frequently (multiply)** treated cohorts. A Poisson regression model was used to assess the evolution between D0 and D80

|  | IRR | [95% Confidence Interval] | | | p-value |
| --- | --- | --- | --- | --- | --- |
| Date of follow-up |  |  |  |  |  |
| Day 0 | 1 |  |  |  |  |
| Day 80 | 0.75 | 0.37 | - | 1.51 | 0.418 |
| Study group |  |  |  |  |  |
| Control | 1 |  |  |  |  |
| Multiply treated | 0.36 | 0.17 | - | 0.76 | 0.007 |
| Date x Study group |  |  |  |  |  |
| Day 0 x Control | 1 |  |  |  |  |
| Day 80 x Multiply treated | 1.03 | 0.37 | - | 2.89 | 0.949 |
|  |  |  |  |  |  |
| Age | 0.98 | 0.96 | - | 1.00 | 0.062 |
| Number of palpable nodules | 1.06 | 0.92 | - | 1.23 | 0.419 |
|  |  |  |  |  |  |
| Anatomic site (nodulectomy) |  |  |  |  |  |
| Head and upper limbs | 1 |  |  |  |  |
| Thorax | 0.47 | 0.18 | - | 1.22 | 0.120 |
| Iliac crests | 0.95 | 0.43 |  | 2.07 | 0.889 |
| Greater trochanters | 0.87 | 0.38 | - | 2.01 | 0.749 |
| Knees and legs | 0.73 | 0.30 | - | 1.77 | 0.486 |
|  |  |  |  |  |  |
| Number of female worms | 1.25 | 1.04 | - | 1.49 | 0.015 |
| Number of male worms | 1.40 | 1.15 | - | 1.71 | 0.001 |
|  |  |  |  |  |  |
| Constant | 0.64 | 0.15 | - | 2.67 | 0.544 |
|  |  |  |  |  |  |
| Random-effect parameters |  |  |  |  |  |
| Nodule level | 3.24 | 2.96 | - | 3.55 |  |

The evaluation of the uterine content was done from 15 μl of the homogenized suspension resulting from the crushing of each female worm. We expressed the numbers of embryos using this volume (15 µl) as arbitrary unit.
