# Supplementary material for: Reproductive Status of Onchocerca volvulus after Ivermectin Treatment in an Ivermectin-Naïve and a Frequently Treated Population from Cameroon
Source: PLoS Negl Trop Dis. 2014 Apr 24;8(4):e2824. doi: 10.1371/journal.pntd.0002824 (PMC3998936; doi:10.1371/journal.pntd.0002824)
Supplement: Text S1 — Procedure for the digestion of nodules to isolate Onchocerca volvulus worms. (DOC) [file pntd.0002824.s005.doc]

**Supporting information**

**Text S1.doc: Procedure for the digestion of nodules to isolate *Onchocerca volvulus* worms**

Nodules stored in liquid nitrogen were thawed between 11:00am and 12:00pm the day before the worm dissection, weighed with 1 mg precision and incubated in the culture medium 199 containing type I collagenase (SIGMA®, Aldrich Co., Oakville, ON, Canada) at a final concentration of 2.25 mg/ml, following the procedure described below:

- nodules whose weight was > 0.500 g were directly incubated in a water bath set at 37°C until 8:00pm. The temperature of the water bath was then lowered to 35°C for overnight incubation until the next day at 8:30am.
- nodules whose weight ranged between 0.300 g and 0.500 g were first incubated on the lab bench (temperature around 25°C) until 8:00pm and then incubated overnight in a water bath set at 35°C until the next day at 8:30am.
- nodules whose weight was between 0.200 g and 0.300 g were incubated on the lab bench (temperature around 25°C) until the next day at 8:30am.
- nodules whose weight was comprised between 0.090 g and 0.200 g were first stored in a refrigerator (temperature around 4°C) until 8:00pm and then incubated overnight on the lab bench (temperature around 25°C) until the next day at 8:30am.
- nodules whose weight was < 0.090 g were stored in a refrigerator (temperature around 4°C) until the next day at 8:30am.

In all cases, the status of tissue digestion of each nodule was checked before living the laboratory (8:00pm) and upon arrival in the laboratory the following day at 8:30am. The incubation process was continued at 37°C (with regular checking) for nodules for which the digestion was not completed.
