# Supplementary material for: Reproductive Status of Onchocerca volvulus after Ivermectin Treatment in an Ivermectin-Naïve and a Frequently Treated Population from Cameroon
Source: PLoS Negl Trop Dis. 2014 Apr 24;8(4):e2824. doi: 10.1371/journal.pntd.0002824 (PMC3998936; doi:10.1371/journal.pntd.0002824)

**Supporting information**

**Text S2.doc: Formulation of the mathematical model** **used for data analyses**

The model can be expressed as follow:


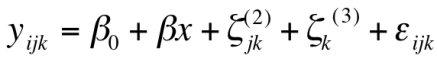
 (Equation 1)

where:

-
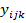
represents the status (productive/not productive) of worm *i* contained in nodule *j* from individual *k*;

- *β_0_* and *βx* are respectively the intercept and the vector of covariates (here the only covariate is « belonging to the control group » *vs* « belonging to the multiply treated one »);

- represents the random intercept parameter for nodule *j* in individual *k*;

-is the random intercept parameter for individual *k*;

-|~ *N* (0,*Ψ*^(2)^),~ *N* (0,*Ψ*^(3)^) and *ε_ijk_* |,, ~ *N* (0, θ).

We assume that the random effects andare independent from each other and from one cluster to another (see Diagram below), we also assume that is independent from one worm to another.


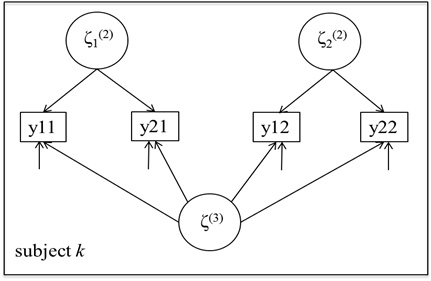

Supplement: Text S2 — Formulation of the mathematical model used for data analyses. (DOC) [file pntd.0002824.s006.doc]
